# GABA_B_ Receptors Tonically Inhibit Motoneurons and Neurotransmitter Release from Descending and Primary Afferent Fibers

**DOI:** 10.3390/life13081776

**Published:** 2023-08-20

**Authors:** Ximena Delgado-Ramírez, Nara S. Alvarado-Cervantes, Natalie Jiménez-Barrios, Guadalupe Raya-Tafolla, Ricardo Felix, Vladimir A. Martínez-Rojas, Rodolfo Delgado-Lezama

**Affiliations:** 1 Department of Physiology, Biophysics and Neuroscience, Centre for Research and Advanced Studies of the National Polytechnic Institute (Cinvestav), Avenida IPN 2508, Col. Zacatenco, Mexico City 07360, Mexico; 2 Department of Cell Biology, Cinvestav, Mexico City 07360, Mexico

**Keywords:** GABA_B_ receptors, spinal cord, dorsolateral funiculus afferents, motoneurons

## Abstract

Motoneurons receive thousands of excitatory and inhibitory synapses from descending tracts and primary afferent fibers. The excitability of these neurons must be precisely regulated to respond adequately to the requirements of the environment. In this context, GABA_A_ and GABA_B_ receptors regulate motoneuron synaptic strength. GABA_A_ and GABA_B_ receptors are expressed on primary afferent fibers and motoneurons, while in the descending afferent fibers, only the GABA_B_ receptors are expressed. However, it remains to be known where the GABA that activates them comes from since the GABAergic interneurons that make axo-axonic contacts with primary afferents have yet to be identified in the descending afferent terminals. Thus, the main aim of the present report was to investigate how GABA_B_ receptors functionally modulate synaptic strength between Ia afferent fibers, excitatory and inhibitory descending fibers of the dorsolateral funiculus, and spinal motoneurons. Using intracellular recordings from the spinal cord of the turtle, we provide evidence that the GABA_B_ receptor antagonist, CGP55845, not only prevents baclofen-induced depression of EPSPs but also increases motoneuron excitability and enhances the synaptic strength between the afferent fibers and motoneurons. The last action of CGP55845 was similar in excitatory and inhibitory descending afferents. Interestingly, the action of baclofen was more intense in the Ia primary afferents than in the descending afferents. Even more, CGP55845 reversed the EPSP depression induced by the increased concentration of ambient GABA produced by interneuron activation and GABA transporter blockade. Immunofluorescence data corroborated the expression of GABA_B_ receptors in the turtle’s spinal cord. These findings suggest that GABA_B_ receptors are extrasynaptic and tonically activated on descending afferent fibers and motoneurons by GABA released from astrocytes and GABAergic interneurons in the cellular microenvironment. Finally, our results also suggest that the antispastic action of baclofen may be due to reduced synaptic strength between descending fibers and motoneurons.

## 1. Introduction

The remarkable variety of behaviors that result from brain activity becomes evident through movements coordinated by motoneurons. Therefore, exquisite control of these cells’ excitability is required to activate the minimum necessary to meet the motor demands. Motoneurons receive many monosynaptic inputs from the ventromedial cord (MF), the reticular formation (RF), the vestibular nucleus (VN), and the dorsolateral funiculus (DLF), which contain rubrospinal and propriospinal fibers that elicit excitatory postsynaptic potentials (EPSPs) in flexor motoneurons, inhibitory postsynaptic potentials (IPSPs), and/or EPSPs in extensor motoneurons and primary afferents [1,2,3,4,5,6,7]. Therefore, for the motor activity to be completed accurately, the synaptic strength of motoneurons must be precisely regulated at both the pre- and postsynaptic levels. On the other hand, it is well known that the γ-aminobutyric acid (GABA) is the primary inhibitory neurotransmitter of the nervous system, which acts through GABA_A_ ionotropic and GABA_B_ metabotropic receptors [8]. Both receptors are expressed in motoneurons and primary afferent fibers, whereas descending afferent fibers contacting motoneurons only express GABA_B_ receptors [1,6]. Interestingly, in descending fibers, the function of GABA_B_ receptors has been elucidated using its agonist, baclofen. In this condition, EPSPs recorded in motoneurons are depressed without affecting the input resistance and membrane time constant [1,3,6]. Therefore, in these synapses, the action of baclofen occurs at the presynaptic level by inhibiting the release of the transmitter.

Likewise, anatomical and functional evidence indicates that Ia fibers make GABAergic axo-axonic contacts that activate synaptic GABA_A_ receptors that mediate short-term presynaptic inhibition associated with primary afferent depolarization (PAD), which is abolished by the application of an antagonist of the GABA_A_ receptors [5,9,10]. It has been shown that sustained activation of GABAergic interneurons produces long-term inhibition that is removed by the blockade of GABA_B_ receptors, activated by a spillover of GABA from axo-axonic synapses [5], suggesting that these receptors are extrasynaptically located in Ia fibers. However, these axo-axonal contacts have not been detected in the descending afferent fibers [1]; therefore, the origin of the GABA that functionally activates the GABA_B_ receptors is presently unknown.

In addition, it has been reported that environmental GABA can tonically activate GABA_B_ receptors in primary afferent fibers, depressing EPSPs [7,11,12]. Also, neither baclofen nor CGP affects the input resistance of postsynaptic neurons [7,12], suggesting that presynaptic GABA_B_ receptors mediate the effects on EPSPs. However, when activated by baclofen, GABA_B_ receptors reduce the excitability of dorsal horn neurons and motoneurons [13,14] by inhibiting L-type calcium channels, indicating that GABA_B_ receptors modulate the active properties of these neurons. Thus, the question arises as to how GABA_B_ receptors are functionally activated in motoneurons and descending fibers of the DLF. One source of GABA may be GABAergic interneurons, as reported in Ia fibers [5]. Another possibility is the astrocytes, as observed in the spinal cord’s dorsal horn and cerebellum, where GABA is released via the protein Bestrophin-1 (Best-1) [15,16].

Interestingly, the activity of GABA_B_ receptors is relevant in patients with spasticity. A widely used pharmacological treatment to reduce this condition is the intrathecal application of baclofen. Synaptic strength studies indicate that baclofen has a greater inhibitory action on Ia fibers [1,6], which has led to the proposal that its antispastic action is due to the inhibition of excitatory transmitter release from the primary afferent fibers. However, in patients with spasticity treated with baclofen, it has been observed that the drug did not affect GABA-mediated presynaptic inhibition, suggesting that baclofen may act on motoneurons but not on Ia afferent fibers [17].

Therefore, the main objective of this report was to investigate how GABA_B_ receptors are activated in the DLF terminals and motoneurons and whether these receptors are tonically active. Likewise, we investigated whether GABA_B_ receptors are extrasynaptic and where the GABA that activates these receptors is produced and released.

## 2. Materials and Methods

### 2.1. Preparation

Forty adult turtles (*Trachemys scripta* spp., 15–20 cm carapace length) were anesthetized with pentobarbital (100 mg/kg, i.p.). The plastron was opened, and the blood was flushed by intraventricular perfusion with Ringer solution (~10 °C) of the following composition (in mM): 120 NaCl, 5 KCl, 15 NaHCO_3_, 3 CaCl_2_, 2 MgCl_2_, and 20 glucose, saturated with 2% CO_2_ and 98% O_2_ to attain pH 7.5. The lumbar spinal enlargement was isolated by a laminectomy and cut transversally to obtain 2–3 mm thick slices. The slices were placed in a recording chamber for the experiments and perfused with Ringer solution (20–22 °C). The spinal cord, in continuity with the dorsal and ventral roots, was dissected and cut transversally to obtain two segments to stimulate Ia primary afferent fibers. At the end of the dissection, the animals were euthanized by decapitation. All experimental procedures followed the guidelines set out in the Journal of Physiology for ethical matters [18] and were conducted with the approval of the Cinvestav-IPN Experimental Ethics Committee, following the current Mexican Norm for the Care and Use of Animals for Scientific Purposes. The animals were provided by the National Mexican Turtle Center located in Mazunte, Oaxaca, Mexico, with authorization (DGVS-03821/0907) from the Federal Mexican Government Ministry of Environment and Natural Resources (Secretaría de Medio Ambiente y Recursos Naturales, Semarnat).

### 2.2. Spinal Cord and DRG Immunostaining

Immediately after the dissection, dorsal root ganglia (DRG) and the lumbar enlargement of the turtle spinal cord were fixed in 4% paraformaldehyde in phosphate-buffered saline (PBS) for 24 h and suspended in 10%, 20%, and 30% sucrose at 4 °C in PBS for 24 h. A total of 20 µm DRG and 30 µm spinal cord slices were obtained using a cryostat (CM1520, Leica Microsystems, Wetzlar, Germany). Tissue sections were permeabilized in 0.3% Triton X-100 in PBS for 10 min, placed in 1% SDS in PBS for 5 min, and blocked in 5% Tween20 and BSA 2% in PBS for 2 h. DRG and spinal cord sections were incubated with mouse anti-GABA_B_ receptor 1 (1:200; ab55051, Abcam) and rabbit NeuN (1:500; #12943, Cell Signaling Technology, MA, USA) for 24 h at 4 °C. After washing three times in PBS, sections were incubated with the corresponding secondary antibodies, Alexa Fluor^®^ 488 AffiniPure Donkey Anti-Rabbit IgG (1:200; 711-545-152, Jackson InmunoResearch, PA, USA) and Alexa Fluor^®^ 594 AffiniPure Donkey Anti-Mouse IgG (1:200; 715-585-150, Jackson InmunoResearch), for 2 h. Then, slices were incubated for 20 min with Hoechst (1:2000; H1398, Invitrogen, MA, USA) and mounted with VECTASHIELD^®^ Antifade Mounting Medium (H-1000, Vector Laboratories, CA, USA). Samples were examined using an epifluorescence microscope (BA410E, Motic, Kowloon, Hong Kong) and Image-Pro Premier software. Images were acquired with the 10× and 40× objectives.

### 2.3. Intracellular Recordings

**Figure 1:**
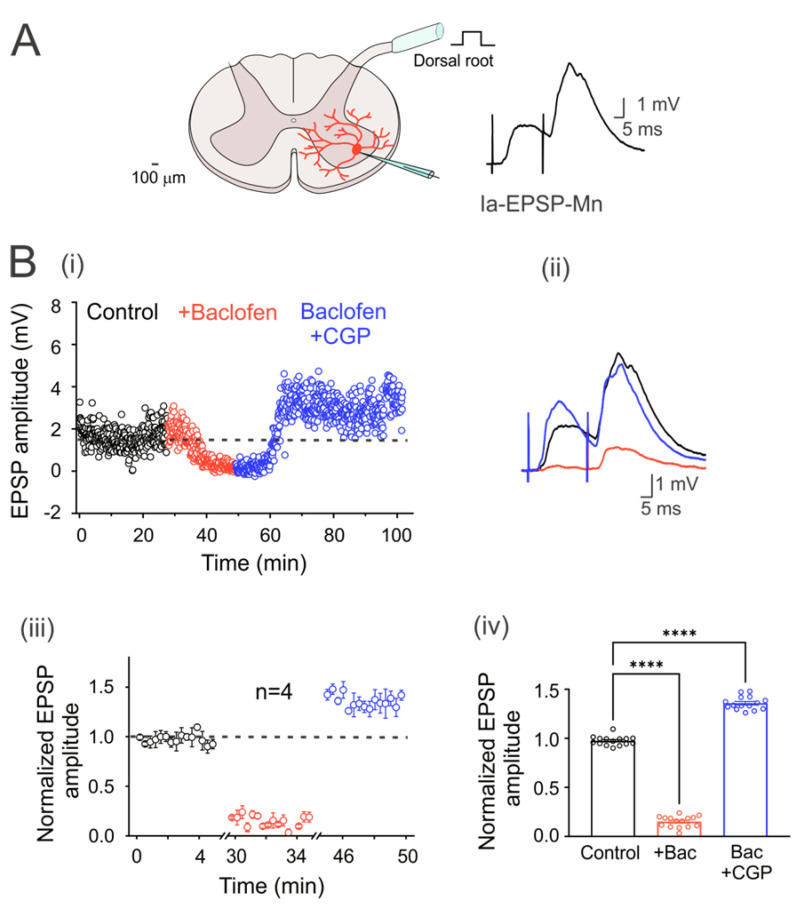
GABA_B_ receptors are tonically active in the Ia primary afferent fibers. (**A**) Schematic showing the cross-section of the lumbar spinal cord with a motoneuron recorded intracellularly (red). The EPSPs were elicited by electrical stimulation (1.3xTh) of the dorsal root. (**B**) (**i**) Time course of the EPSP amplitude recorded in a motoneuron in the control bath solution (2 µM strychnine; black circles), baclofen (red circles), and baclofen plus CGP55845 (blue circles). (**ii**) Representative traces of the EPSPs recorded in the three experimental conditions. (**iii**) Segmented time course of normalized steady-state EPSP amplitude, relative to control values, recorded during the last 5 min in each condition of steady-state response in each condition with respect to the mean control EPSP amplitude. (**iv**) Bar graph summarizing the mean values of the normalized EPSPs shown in (**iii**) for four motoneurons under all experimental conditions. Asterisks denote statistically significant differences in the values of the EPSPs (*p* < 0.0001 by one-way ANOVA).

The input resistance and the postsynaptic potentials (EPSPs and IPSPs) were monitored by applying a negative rectangular current pulse (500 ms duration) every 5 s before the paired-pulse stimulation of the DLF or dorsal root (Figure 1A). The EPSP and IPSP were evoked in motoneurons by electrical stimulation (1.3xTh) of the DLF and the dorsal root every 5 s in the presence of strychnine and 6-Cyano-7-nitroquinoxaline-2, 3-dione (CNQX). Here, it is worth mentioning that IPSPs could be fully discriminated by the AMPA receptor blockade with CNQX (at 20 µM) because the expression of NMDA receptors has not been reported in motoneurons, and therefore, AMPA receptors are considered to be the only ones responsible for mediating EPSPs in these cells [19]. In the first case, a tungsten bipolar electrode (World Precision Instruments, Sarasota, FL, USA) was used. In the second case, the glass pipette suctioned the dorsal root. The tungsten electrode and the glass pipette were connected to a source of constant current (Neuro Data Instruments Corp., TX, USA). The threshold was defined as the minimum stimulus current intensity that elicits a measurable postsynaptic potential (PSP). The PSPs were recorded with an Axoclamp 2B amplifier (Molecular Devices, Union City, CA, USA) with a bandwidth of 0.1 Hz to 10 KHz, digitized at 10 KHz, and stored in a hard disk for off-line analysis.

### 2.4. Drugs

GABA_B_ receptors were activated with baclofen (2 µM) and blocked with CGP55845 (5 µM). Glycinergic receptors were blocked by strychnine (2 µM) and ionotropic glutamatergic AMPA receptors with CNQX (20 µM). Nipecotic acid (100 µM) and CaCCihn-A01 (20 µM) were used to block the activity of GABA transporters and Bestrophin-1 channels, respectively. All drugs used in this study were purchased from Sigma Chemical Company (St. Louis, MO, USA). Baclofen was dissolved in DMSO according to the manufacturer’s instructions and stored at 4 °C. From this initial dilution, aliquots (of 10 mM) were prepared and kept at 4 °C. From these aliquots, fresh working solutions were prepared just before each experiment.

### 2.5. Statistical Analysis

The time course response of the PSP of each motoneuron to the application of the drugs was not the same; therefore, to evaluate the effects for each group of neurons, 4 consecutive EPSPs were recorded every 5 s over 5 min at the steady state for each condition (drug and train) and averaged. These values were normalized to the average control value of each neuron and plotted as a function of time and presented in a bar graph. The action potentials were elicited by 5 s depolarizing current injections for the motoneuron excitability test. Waveform kinetics was determined by differentiating the spike voltage over time (dV/dt), and phase graphs were constructed by plotting the membrane voltage versus the first derivative (mV/ms). The excitability was evaluated by plotting the number of action potentials as the function of supra-threshold intracellular depolarizing current pulses starting from the rheobase current. One-way ANOVA and Student’s *t*-test determined the statistical differences between the means. Means were considered statistically different when *p* < 0.05. Values were presented as the mean ± S.E.M.

## 3. Results

### 3.1. Characterization of Motoneurons

The recorded cells of the spinal ventral horn were classified as motoneurons (*n* = 41) when, after being stimulated with a pulse of intracellular depolarizing current greater than the rheobase (Figure 1A), they presented adaptation of the action potential firing pattern. The average input resistance of these cells was 18 ± 3.2 MΩ (*n* = 41), and the membrane time constant was 19 ± 0.5 ms (*n* = 41), consistent with previously reported values for motoneurons [4,20].

### 3.2. GABA_B_ Receptors Are Tonically Active in Ia Primary Afferent Fibers

**Figure 2:**
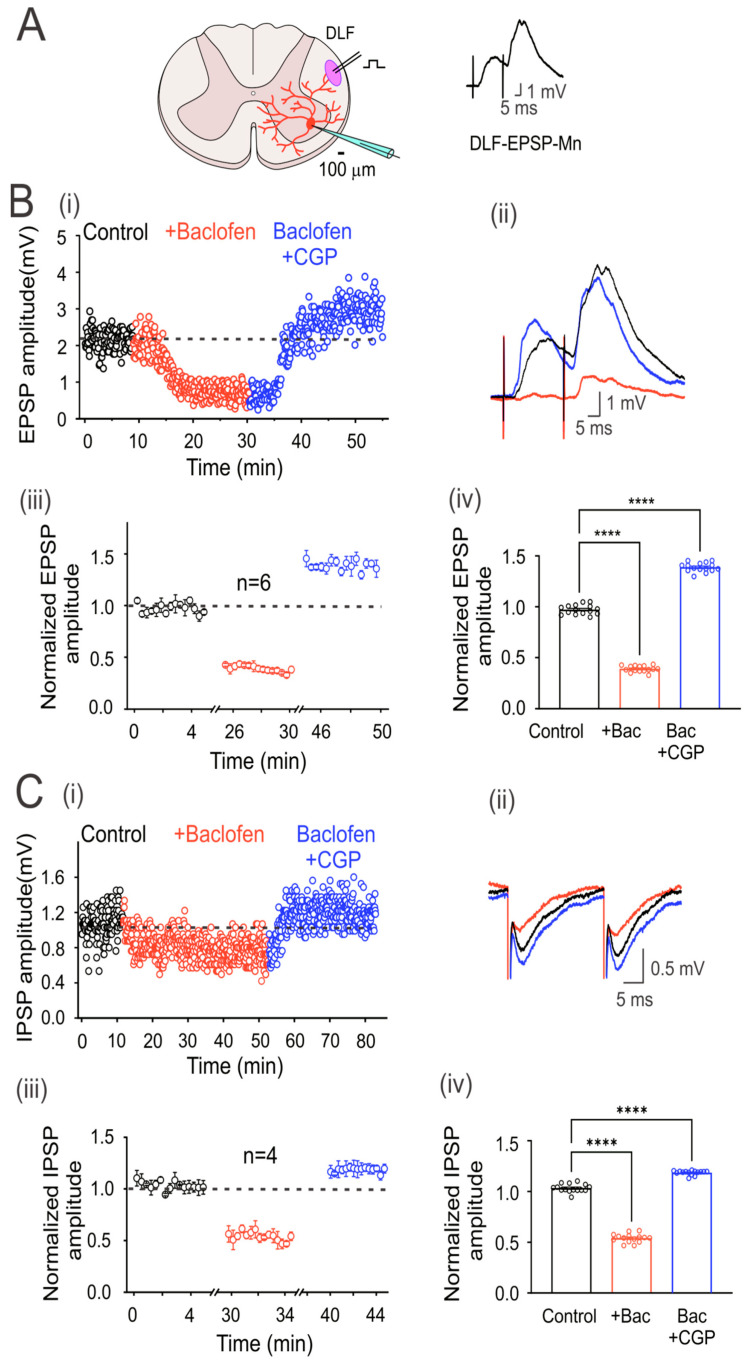
GABA_B_ receptors are tonically active in excitatory and inhibitory DLF fibers. (**A**) Schematic showing the transverse section of the lumbar spinal cord with a motoneuron recorded intracellularly (red). Here and throughout the subsequent outcomes, the EPSP was elicited by the electrical stimulation (1.3xTh) of the DLF. (**B**) Time course of the EPSP recorded in control bath solution (strychnine 2 µM; black circles), baclofen (red circles), and baclofen plus CGP55845 (blue circles) (**i**). (**C**) IPSP was recorded as in (**B**), but CNQX 20 µM was added instead of strychnine in the control bath solution. Representative EPSP (**B**) and IPSP (**C**) traces recorded under their respective conditions are shown (**ii**). Segmented time course of normalized EPSP (**Biii**) and IPSP (**Ciii**) amplitude recorded during 5 min of steady-state response in each condition with respect to the mean control EPSP amplitude. Bar graph showing the mean values of the EPSPs (**Biv**) and IPSPs (**Civ**), recorded over 5 min. Asterisks denote statistically significant differences in the values of the EPSPs and IPSPs (*p* < 0.0001 by one-way ANOVA).

In order to corroborate that turtle Ia primary afferents were presynaptically inhibited by tonic activation of GABA_B_ receptors, as in rats, monosynaptic Ia-EPSPs were evoked in motoneurons by dorsal root stimulation at 1.3xTh every 5 s (Figure 1A). Figure 1(Bi) shows the time course of an evoked Ia-EPSP amplitude in a representative neuron in the presence of baclofen alone or baclofen plus CGP55845. Taking the dashed line as the mean value of the control condition, it can be seen that baclofen produced a depression of the EPSP amplitude, as shown in the representative traces on the right (Figure 2(Bii)). Application of CGP55845 not only reversed EPSP depression but also augmented its amplitude. As can be seen in the graph of normalized values (Figure 1(Biii)) and the corresponding bar graph on the right (Figure 1(Biv)), baclofen depressed the amplitude of the EPSPs by 84.6 ± 0.01% (*n* = 4, ANOVA test; F_(2,42)_ = 1706; *p* < 0.001), while CGP55845 facilitated it by 45 ± 0.02% (*n* = 4, ANOVA test; F_(2,42)_ = 1706; *p* < 0.001), concerning the control value. The input resistance (control 16.5 ± 5.4; baclofen 16.4 ± 4.2 MΩ; *n* = 4, *p* > 0.5) and the time constant (control 19.7 ± 0.8; baclofen 20.2 ± 0.7 ms; *n* = 4, *p* > 0.5) were not affected by the drug treatment. Furthermore, the amplitude ratio increased by 93.8% in the presence of baclofen. Overall, these findings agree with previous studies performed in rats, suggesting a conserved mechanism of presynaptic inhibition mediated by GABA_B_ receptors.

### 3.3. GABA_B_ Receptors Are Tonically Active in the DLF Terminals Synapsing Motoneurons

EPSPs in motoneurons were evoked by electrical stimulation of the DLF at 1.3xTh (0.3 ms) every 5 s (Figure 2A). However, since the DLF stimulation evokes EPSPs and IPSPs, the glycine receptor antagonist strychnine (2 μM) was added to the bathing solution (control) to isolate EPSPs. Figure 2(Bi and Bii) show the time course and averaged traces of evoked DLF-EPSPs in a representative motoneuron, in the control condition and the presence of baclofen alone and baclofen plus CGP55845. As observed with the Ia-EPSPs, baclofen depressed the DLF-EPSPs, while CGP55845 not only reversed depression but also facilitated the EPSPs. As shown in the graph of normalized values (Figure 2(Biii)) and the corresponding bar graph on the right (Figure 2(Biv)), baclofen depressed EPSP amplitude by 59 ± 0.01% (*n* = 6, ANOVA test; F_(2,42)_ = 2204; *p* < 0.001), and CGP55845 facilitated it by 43 ± 0.01% (*n* = 6, ANOVA test; F_(2,42)_ = 2204; *p* < 0.001), concerning the control, in six recorded neurons. On average, the amplitude of the DLF-EPSPs was reduced by baclofen to a lesser extent than the Ia-EPSPs.

Next, to record IPSPs in motoneurons elicited by DLF stimulation, an AMPA receptor antagonist (CNQX, 20 µM) was added to the bath solution to block the EPSPs. Figure 2(Ci) shows the time course of evoked DLF-IPSPs in a representative motoneuron under the control condition, in the presence of baclofen alone, or baclofen plus CGP55845. Figure 2(Cii) shows representative traces of the IPSPs recorded under the three conditions. The addition of CGP55845 had two apparent effects. First, it reversed the action of baclofen, and second, it facilitated the amplitude of DLF-IPSPs. As shown in the normalized value plot (Figure 2(Ciii)) and the corresponding bar graph on the right (Figure 2(Civ)), baclofen depressed the IPSP amplitude by 46 ± 0.01% (*n* = 4, ANOVA test; F_(2,42)_ = 1314; *p* < 0.001), while CGP55845 facilitated it by 18 ± 0.01% (*n* = 4, ANOVA test; F_(2,42)_ = 1314; *p* < 0.001), concerning the control. As observed with the DLF-EPSPs, the DLF-IPSPs were less affected than the Ia-EPSPs.

It should be noted that in all motoneurons recorded, the input resistance (control 14.8 ± 1.7 MΩ; baclofen 15.4 ± 2.4 MΩ; *n* = 10; *p* = 0.83) and the time constant of the membrane (control 18.9 ± 0.2; baclofen 19.2 ± 0.2 ms) were not affected by baclofen, suggesting that the depression of the EPSPs and IPSPs was caused by GABA_B_ receptor-mediated presynaptic inhibition of DLF afferent fiber terminals.

### 3.4. On the Origin of GABA

**Figure 3:**
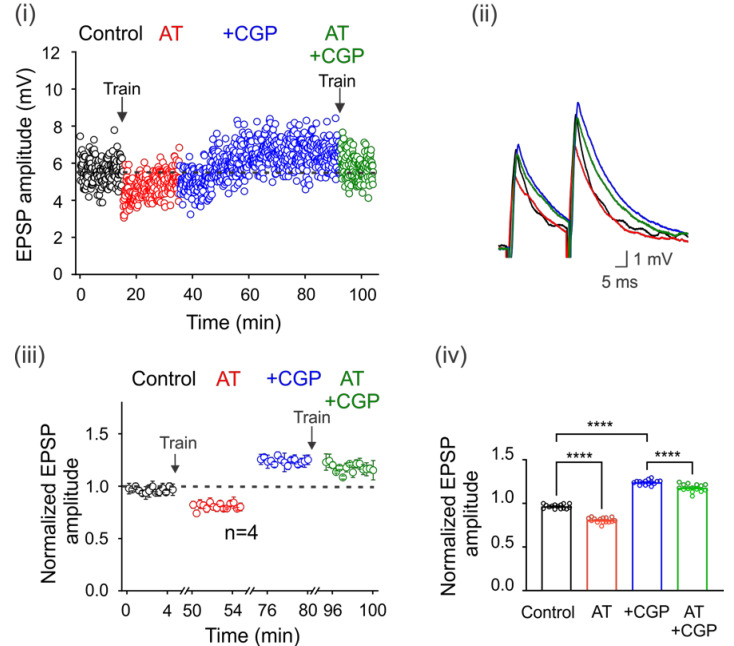
Spillover of GABA tonically activates GABA_B_ receptors. GABA spillover tonically activates GABA_B_ receptors. (**i**) Time course of the EPSPs elicited by DLF stimulation at 1.3xTh in the control bath solution (3 μM strychnine), after a train (AT) of 100 electrical pulses (100 Hz) applied to the spinal cord premotor area, in the presence of CGP55845 and again after a second train of pulses. (**ii**) Superimposed traces from representative EPSPs recorded under the four experimental conditions are shown. The color of the individual traces corresponds to the conditions described in (**ii**). Normalized EPSP amplitude was recorded over 5 min at steady state in each condition relative to the mean EPSP amplitude in the control. The number of independent motoneurons evaluated under each condition is shown below the symbols in (**iii**). (**iv**) Bar graph showing the mean value of the EPSPs recorded for 5 min for four motoneurons. Asterisks indicate statistically significant differences (*p* < 0.0001 by one-way ANOVA).

It is well known that motoneurons are innervated by GABAergic and glycinergic interneurons. Therefore, we next decided to investigate whether electrical stimulation of premotor interneurons with a train of 100 pulses at 100 Hz could increase ambient GABA concentration, eventually activating GABA_B_ receptors. Figure 3i shows the time course of EPSP amplitude in a representative motoneuron in the control condition after one train of activating pulses (AT) and after a second train in the presence of CGP55845. Interestingly, the depression of the EPSPs produced by the first train was reversed and facilitated with CGP55845, which prevented the depression after the second train (Figure 3i,ii). As shown in the normalized and averaged bar graphs, stimulation resulted in the depression of the DLF-EPSPs by 18 ± 0.01% (*n* = 4, ANOVA test; F_(3,56)_ = 598.5; *p* < 0.001) for more than 5 min. However, when CGP55845 was applied, DLF-EPSPs were facilitated by 24 ± 0.01% (*n* = 4, ANOVA test; F_(3,56)_ = 598.5; *p* < 0.001), and the second-train decrease was also prevented (Figure 3iii,iv). Considering that DLF terminals do not express GABA_A_ receptors and only express GABA_B_ receptors, these results indicate that the pulse train produced GABA release from GABAergic interneurons, increasing the number of active GABA_B_ receptors expressed on DLF terminals. This conclusion is supported by the observation that in the presence of CGP55845, the amplitude of the basal EPSPs was restored and facilitated, preventing a decrease in amplitude after a second train of pulses.

### 3.5. Regulation of Ambient GABA Concentration with GABA Transporters

**Figure 4:**
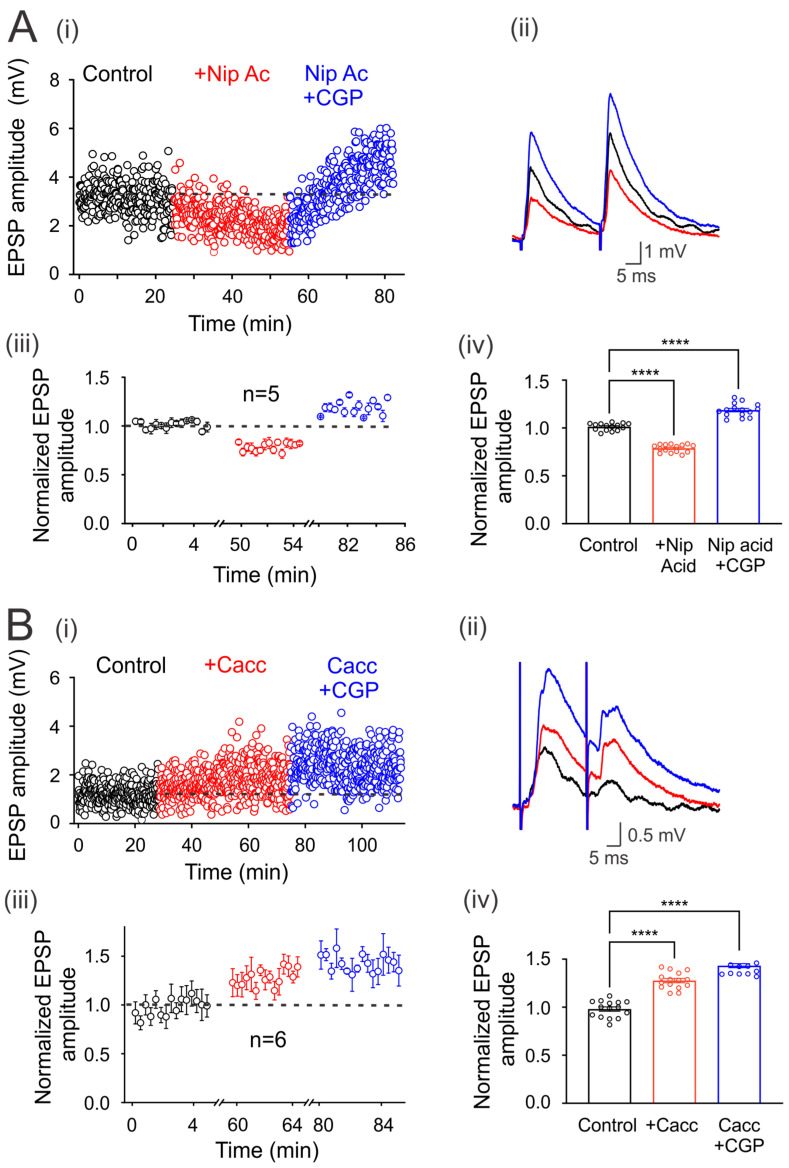
Modulation of environmental GABA concentration. (**Ai**) Time course of the EPSPs evoked every 5 s by DLF stimulation at 1.3xTh in the control bath solution (3 µM strychnine), in the presence of nipecotic acid (NA) and NA plus CGP55845 (**i**). (**Bi**) Time course of the EPSPs evoked every 5 s by DLF stimulation at 1.3xTh in the control bath solution (3 µM strychnine), in the presence of CaCCinh (Cacc) and Cacc plus CGP55845 (**i**). (**A**) and (**B**) (**ii**) show the traces of representative EPSPs recorded in the different conditions. (**iii**) Amplitude of the normalized EPSPs recorded for 5 min at steady state in each experimental condition to the mean control amplitude. (**iv**) Bar graph showing mean values of EPSPs recorded over 5 min for six motoneurons in each condition. Asterisks indicate statistically significant differences (*p* < 0.0001 by one-way ANOVA).

It is also known that neurons and astrocytes express transporters that can uptake synaptically released GABA. Therefore, we next decided to investigate whether the blockade of these transporters may affect environmental GABA that tonically activates GABA_B_ receptors. Figure 4Ai shows that the application of nipecotic acid (100 µM) decreases the amplitude of EPSPs compared to the control condition in one representative motoneuron. This effect was reversed and facilitated by adding CGP55845 to the bath solution (Figure 4(Ai,ii)). A similar effect was observed in five neurons that presented a mean EPSP depression of 21 ± 0.01%, concerning the control (*n* = 5, *p* < 0.05; Student’s *t*-test). As previously observed, CGP55845 reversed its decrease and facilitated EPSPs by 19 ± 0.02%, concerning the control values (*n* = 5, ANOVA test; F_(2,42)_ = 226.7; *p* < 0.001) (Figure 4(Aiii,iv)). These results show that GABA uptake by transporters may regulate the environmental neurotransmitter concentration and, therefore, the number of tonically activated GABA receptors.

### 3.6. GABA May Be Released through Best-1 Channels

It has been reported that extrasynaptic GABA_A_ receptors are tonically active by ambient GABA released by astrocytes through the Best-1 protein channel [16]. Therefore, in order to know whether the ambient GABA that activates the extrasynaptic GABA_B_ receptors (expressed in the DLF-motoneuron synapse) is released through Best-1 channels, we decided to block this channel with CaCCinh, an antagonist of the Best-1 protein. As is shown in Figure 4(Bi–iv), the EPSP amplitude was facilitated by CaCCinh (20 µM) in 27 ± 0.02%, concerning control (*n* = 6, *p* < 0.001, Student’s *t*-test) while, CGP55845 produced an additional facilitation of 15 ± 0.021% (*n* = 6, ANOVA test; F_(2,42)_ = 107.2; *p* < 0.001). Therefore, the facilitation of the EPSPs could be due to the Best-1-mediated decrease in the ambient GABA concentration, resulting in a lower amount of tonically active GABA_B_ receptors.

### 3.7. GABA_B_ Receptors Tonically Inhibit Motoneuron Excitability

**Figure 5:**
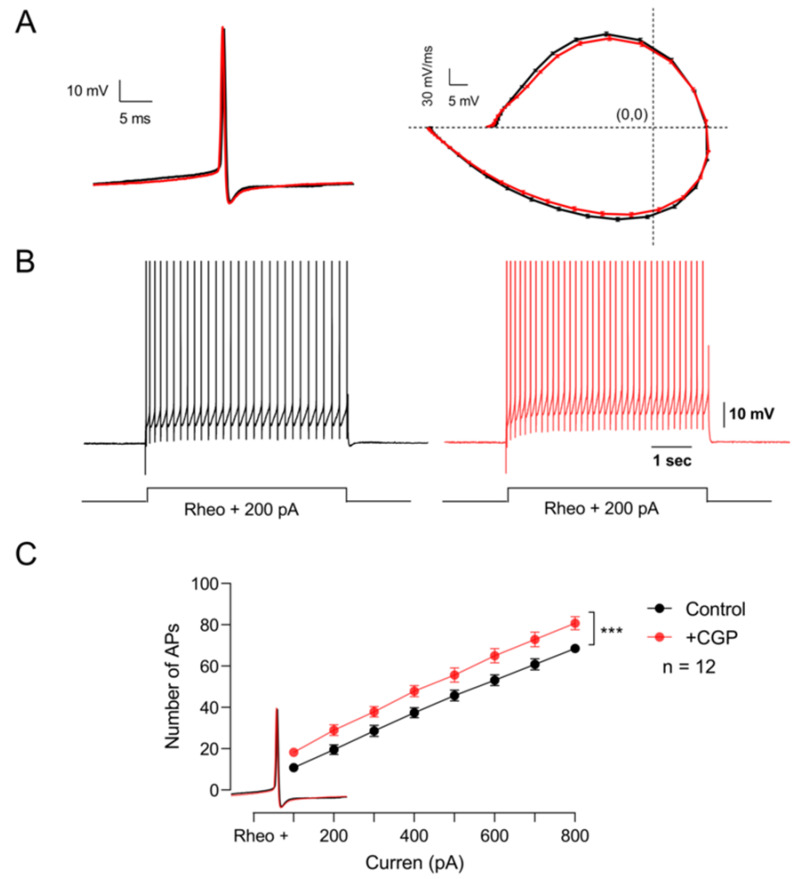
GABA_B_ receptors tonically depress the excitability of motor neurons. (**A**) On the left, representative action potentials recorded in the control bath solution (black) and the presence of CGP55845 (red). The phase plot of membrane voltage versus the first derivative (mV/ms) of the action potential is shown on the right. (**B**) Firing pattern of the action potential elicited with the same depolarizing current in the control condition (black) and the presence of CGP55845 (red). (**C**) Graph of the number of action potentials as a function of the injection of depolarizing intracellular current recorded in the control bath solution (black) and the presence of CGP55845 (red). Asterisks indicate statistically significant differences (*p* < 0.001 by Student’s *t*-test).

Experimental evidence indicates that baclofen application does not affect the passive properties of motoneurons. However, Svirskis and Hounsgaard (1998) showed that activation of GABA_B_ receptors with baclofen inhibits an inward calcium current through L-type channels [14]. Therefore, we next decided to investigate whether these receptors are modulating the firing pattern of these cells. To this end, excitability was determined by applying supra-threshold depolarizing current pulses under control conditions and in the presence of CGP55845. The results of these series of experiments show that the blockade of GABA_B_ receptors with CGP55845 did not modify the kinetics of the single action potential (Figure 5A), but significantly increased the firing frequency (Figure 5B,C) (*n* = 12, Student’s *t*-test; t_(7)_ = 17.4; *p* < 0.001). These results suggest that GABA_B_ receptors expressed in motoneurons are tonically decreasing the excitability of motoneurons.

### 3.8. GABA_B_ Receptor Expression in the Spinal Cord and Dorsal Root Ganglia of the Turtle

**Figure 6:**
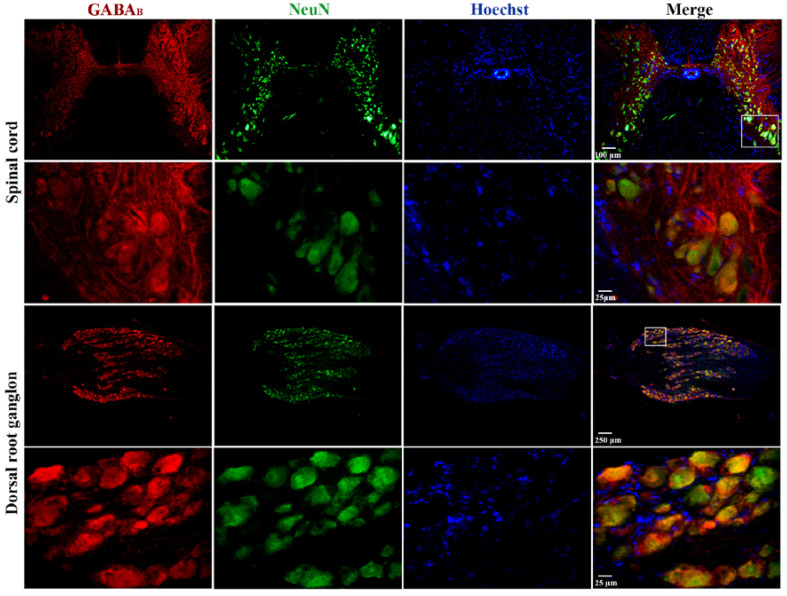
Expression of GABA_B_ receptors in the spinal cord and dorsal root ganglia of the turtle. Images shown correspond to cross-sections of the spinal cord and DRG immunostained with antibodies against GABA_B_ receptors in red, neuronal nuclei (NeuN) in green, and nuclei (Hoechst) in blue. In both cases, the images in the upper row were obtained with the 10× objective, and those in the lower row were obtained with the 40× objective. A magnified image of a selected region is also shown, as indicated. The combined image suggests colocalization (yellow/orange) of GABA_B_ receptors in neuronal cell bodies. It should be noted that Hoechst spots might not overlap (colocalize) with the fluorescence of anti-NeuN antibodies because the light intensity to activate Hoechst fluorescence in large cells is not strong enough to see it in its entirety, as if it occurs in neurons of small size. For this same reason, the nuclei of satellite (small) glial cells surrounding the soma of neurons in the DRG stain adequately. In addition, negative controls are presented as a Supplemental Figure S1.

Finally, immunohistochemical staining was performed on cross-sections of the spinal cord and dorsal root ganglia (DRG) to determine the expression of GABA_B_ receptors in these regions. The results of this analysis show that GABA_B_ immunostaining is more prominent in the dorsal horn than in the ventral horn of the spinal cord (upper panel of Figure 6). Interestingly, in the ventral horn (lower panels of Figure 6), the signal was evident in the largest cells that, due to their size, may correspond to motoneurons. In Figure 6, immunostaining for GABA_B_ in the DRG (upper panel) is present in almost all neurons regardless of size, which is best appreciated in the magnification of the boxed region shown in the bottom panels of Figure 6.

## 4. Discussion

In the present report, we show that the GABA_B_ receptors are tonically activated by ambient GABA, inhibiting the excitability of motoneurons and the transmitter release from excitatory and inhibitory DLF terminals synapsing motoneurons. Likewise, we corroborated that these receptors tonically inhibit Ia afferent fibers. We also show that the number of tonically activated GABA_B_ receptors depends on the ambient GABA concentration, which is determined by its release from interneurons and astrocytes. Therefore, extrasynaptic GABA_B_ receptors may have a relevant role in motor control.

### 4.1. GABA_B_ Receptors Tonically Inhibit Transmitter Release from DLF Terminals and Ia Afferents

The role of GABA_B_ receptors in modulating the synaptic strength of excitatory DLF terminals and motoneurons was investigated in the presence of strychnine to block the inhibitory potentials evoked by the DLF terminals of propriospinal origin [2]. In these experimental conditions, baclofen was found to decrease the EPSPs and IPSPs elicited by stimulation of the DLF and Ia fibers, respectively. In both cases, CGP55845 prevented the decrease and facilitated the EPSPs and IPSPs. Notably, the observed effects on the EPSPs and IPSPs did not alter the motoneurons’ input resistance or time constant. This finding agrees with previous reports in Ia, Aβ, Aδ, and C afferent fibers, where a blockade of GABA_B_ receptors facilitated the EPSPs evoked in motoneurons and dorsal horn neurons [7,11,12].

Likewise, the blockade of GABA_B_ receptors in primary afferent fibers and DLF terminals did not affect miniature EPSCs (mEPSCs), miniature IPSCs (mIPSCs), or membrane conductance [6,11]. Before our work, the function of these receptors in the afferent fibers of descending tracts, such as the ventromedial, dorsal, and dorsolateral funiculus, had been evidenced only by their activation with baclofen, producing a significant inhibition of transmitter release [1,3,4,6].

Interestingly, in excitatory DLF afferents, GABA_B_ receptors may act by inhibiting Ca^2+^ channels of the N- and P/Q-type via the activation of Gi/o proteins [21]. On the other hand, the expression of GABA_A_ receptors in the DLF terminals has been underestimated because, unlike Ia fibers, the vestibulospinal and reticulospinal terminals that synapse with motoneurons appear not to be under the presynaptic control of muscle-type afferents I and II [1]. Moreover, there is no evidence of axo-axonic GABAergic synapses in these afferents [22]. Therefore, DLF terminals may only express GABA_B_ receptors located at extrasynaptic sites. Another relevant result of our work is that the blockade of the GABA_B_ receptors produced a facilitation of EPSPs and IPSPs, which indicates that these receptors, as in primary afferent fibers, are tonically active by the ambient GABA.

### 4.2. The Cellular Origin of GABA

Though the descending afferent fibers express GABA_B_ receptors, GABAergic axo-axonal contacts, such as those in primary afferents that release GABA, have not yet been identified. Here, we show that the simultaneous stimulation of interneurons in the premotor region produces a depression of EPSPs that is prevented by applying CGP55845, suggesting the involvement of GABA_B_ receptors. This further suggests that synaptically released GABA must have diffused from the synaptic gap to reach extrasynaptic receptors. This result is consistent with what has been described for CA3 pyramidal cells of the hippocampus, where extrasynaptic GABA_B_ receptors are activated by GABA released by the simultaneous activation of neighboring interneurons [23]. Likewise, in the spinal cord, activation of these receptors expressed in the primary afferents was produced by tetanic stimulation of the hindlimb flexor muscles, leading to long-lasting presynaptic inhibition of Ia fibers, which is prevented with the application of CPG [5]. Thus, in the spinal cord, diffusion of synaptically released GABA can regulate synaptic strength between primary and descending afferent fibers with motoneurons, which is relevant to motor control. Activation of GABA_B_ receptors by GABA diffusion indicates that these receptors are extrasynaptic. Consequently, the functioning of neural networks in the spinal cord, just as it occurs in the hippocampus, requires the action of GABA released from the synaptic and diffusion systems. The diffusion of neurotransmitters is known as spillover and, together with its related receptors, is called the neurotransmitter diffusion system [24].

The depression of the EPSPs produced by the blockade of the GABA transporters and their reversal and facilitation by CGP55845 suggest that in the process, more GABA_B_ receptors were activated than those already tonically active, which, when blocked, facilitated the EPSPs in response to the increase in GABA concentration. This enhanced action of GABA produced by blocking GABA uptake has been reported in neurons of the spinal cord, cerebellum, cortex, and hippocampus [25,26]. Particularly in the spinal cord, the blockade of GABA transporters with nipecotic acid prolongs the neuronal response to the iontophoretic application of GABA [25]. Therefore, GABA transporters expressed in neurons and glial cells may play an important role in the uptake of neurotransmitters in the synaptic cleft and in controlling its extrasynaptic concentration [27].

Another potential source of GABA is astrocytes. EPSP facilitation in the DLF in the presence of CaCCinh, a Best-1 channel blocker, indicates a reduction in active GABA_B_ receptors due to decreased ambient GABA concentration. This is consistent with the reduction in the tonic currents recorded in cerebellar granule cells in the presence of the Best-1 antagonist [16]. Interestingly, GABA and Best-1 channels have previously been shown to be expressed in Bermann glia and lamellar astrocytes adjacent to granule cell bodies [16]. In addition, glutamate has been shown to induce GABA release from astrocytes in the spinal cord, as recorded using HEK293 cells heterologously expressing GABA receptors as GABA sensors [15]. Likewise, GABA transporters expressed in neurons and glial cells regulate the extracellular concentration of GABA by capturing and releasing it, as occurs in the Bermann glia [27,28].

### 4.3. The Motoneuron Excitability Is Controlled Tonically by GABA_B_ Receptors

In all motoneurons recorded, baclofen depressed the EPSPs and IPSPs evoked by DLF and Ia afferent fibers without affecting their passive properties. The same action of baclofen was reported in the recordings of EPSPs evoked in motoneurons by activation of descending afferent fibers and Ia [1,5,6] and those recorded in dorsal horn neurons of the spinal cord evoked by activation of the primary afferents Aβ, Ad, and C [7,11,12]. Furthermore, it has been shown that the size of the quantum of miniature potentials did not change, indicating that the action of baclofen was at the presynaptic level [6]. However, in these reports, the active properties of postsynaptic neurons were not evaluated [6,7,11,12]. Our findings show that in motoneurons, GABA_B_ receptors are tonically reducing excitability, since blockade with CGP55845 increases the number of action potentials. This result suggests that, as in afferent fibers, GABA_B_ receptors are tonically active in motoneurons, reducing their excitability due to the inhibition of the L-type calcium current. This action of GABA_B_ receptors was reported by Svirskis and Hounsgaard, who showed that in motoneurons, the activation of the inward calcium current through L-type channels was inhibited in the presence of baclofen [14].

On the other hand, immunofluorescence assays have shown that GABA_B_ receptors are located in both the dorsal and ventral horns of the turtle spinal cord, with greater expression dorsally and 60–90% lower density in laminae IX and X than in the superficial laminae of the rat spinal cord [29]. Probably the high density of GABA_B_ receptors in the dorsal horn represents its expression not only in the soma and neuropil of interneurons but also in primary afferents of the Aδ-type and C-type [30]. The positively stained cells in the ventral horn may correspond to motoneurons due to their size and localization [31]. In the DRG, we found a high expression level of GABA_B_ receptors in all cells regardless of size. This result is similar to that reported for the DRG in rats [29]. Therefore, the images indicate that GABA_B_ receptors are expressed in the postsynaptic neurons throughout the spinal cord, where they control its excitability.

### 4.4. GABA_B_ Receptors and Spasticity

Spasticity is a condition present in patients with injury of the brain and spinal cord, defined as a motor disorder with a velocity-dependent increase in tonic stretch reflexes with exacerbated tendon jerks caused by hyperexcitability of the stretch reflex, as a component of the upper motoneuron syndrome [32,33]. For a long time, baclofen has been used to reduce spasticity, although it is presently unknown which neuronal element is acting.

Based on the evidence showing that the main action of baclofen is to decrease transmitter release from Ia afferent and descending afferent fibers that synapse with motoneurons [1], it has been proposed that the antispastic action of baclofen is due to the presynaptic inhibition of Ia fibers. However, it has also been shown that, in patients with spasticity, neither of the two tests of presynaptic inhibition, postactivation depression, or reciprocal disynaptic inhibition of the soleus H reflex were affected by baclofen administration. In addition, primary afferent action potentials did not change after baclofen administration. However, in both cases, baclofen depressed the H reflex. Based on these results, the antispastic action of baclofen could be attributed to a decrease in motoneuron excitability [17].

Interestingly, in all studies performed on animal preparations, the reported effect of baclofen is the inhibition of transmitter release from primary Ia afferents and excitatory descending afferents of the ventromedial and dorsolateral funicles [1,3,4,7,21] without affecting the passive properties of motoneurons. In these reports, only the passive properties were explored, either by recording the input resistance or by determining the time constant of the EPSP decay phase, without studying the action of baclofen on the active properties of motoneurons. Our results indicate that GABA_B_ receptors tonically inhibit motoneuron excitability. This could be explained by the inhibition of L-type calcium currents by baclofen [14]. This postsynaptic action of baclofen was also observed in the dorsal horn neurons of the spinal cord, where activation of GABA_B_ receptors inhibits the wind-up and potential plateau that underlies a sustained burst of action potentials [13]. Our results support the idea that the antispastic action of baclofen may be produced by the reduction in motoneuron excitability due to the inactivation of the presynaptic N- and P/Q-type calcium channels in the descending excitatory fibers [21] and the postsynaptic L-type calcium channels expressed in motoneurons.

In conclusion, this paper shows evidence that GABA_B_ receptors are tonically activated by environmental GABA, thereby reducing synaptic strength between DLF afferent fibers and motoneurons by inhibiting transmitter release and postsynaptic excitability. Furthermore, we found that the release from interneurons and astrocytes determines the environmental GABA concentration.

## Data Availability

Derived data supporting the findings of this study are available from the corresponding authors upon request.

## Supplementary Materials

The following supporting information can be downloaded at: https://www.mdpi.com/article/10.3390/life13081776/s1, Figure S1: Negative control of immunofluorescence experiments of the expression of GABA_B_ receptors in the turtle’s spinal cord and dorsal root ganglia. (A) Images shown correspond to cross-sections of the spinal cord (A) and DRG (B) immunostained without primary antibodies against GABA_B_ receptors and neuronal nuclei (NeuN), but in the presence of the fluorescent DNA dye Hoechst (blue signal). The images in the upper and lower rows were obtained with the 4× and 10× objectives, respectively.
