# Supplementary material for: GABAB Receptors Tonically Inhibit Motoneurons and Neurotransmitter Release from Descending and Primary Afferent Fibers
Source: Life (Basel). 2023 Aug 20;13(8):1776. doi: 10.3390/life13081776 (PMC10456076; doi:10.3390/life13081776)
Supplement: Supplementary file 1 [file life-13-01776-s001.zip › life-2503714-supplementary.pdf]

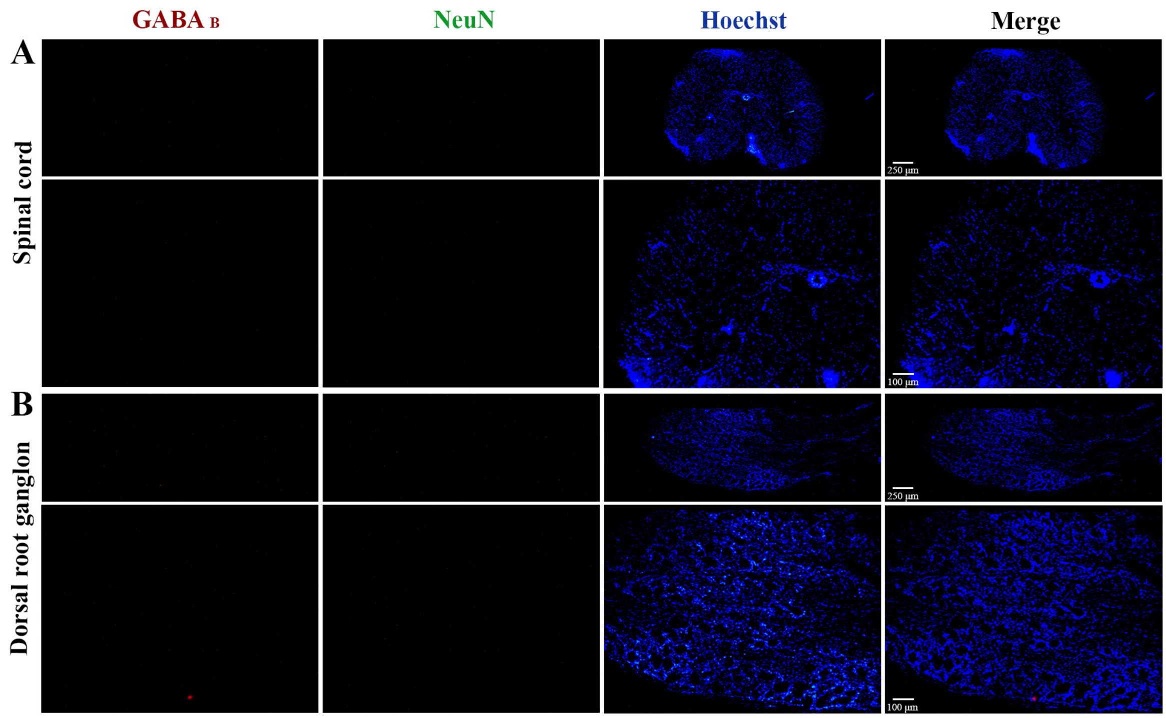

Supplemental Figure S1. Negative control of immunofluorescence experiments of the expression of GABA$_B$ receptors in the turtle's spinal cord and dorsal root ganglia. A) Images shown correspond to cross-sections of the spinal cord (A) and DRG (B) immunostained without primary antibodies against GABA$_B$ receptors and neuronal nuclei (NeuN), but in the presence of the fluorescent DNA dye Hoechst (blue signal). The images in the upper and lower rows were obtained with the 4X y 10X objectives, respectively.
